# Editorial Extracts and Gleanings

**Published:** 1874-02

**Authors:** C. H. Tidd

**Affiliations:** Ohio


### EXTRACTS AND GLEANINGS

BY C. H. TIDD, M.D., OF OHIO.

*Cerebal Paresis Treated with Phosphorous.*—Dr. G. Tempini attributes this disease to an excessive waste of protagon, ($C^{222}$ $H^{240}$, N 4, Ph $O^{44}$,) generally the result of over use of the cerebral or reproductive functions. He prescribes five centigrammes of phosphorous, to be dissolved in some ether, and mixed with enough powder and extract of liquorice to make a mass which may be divided into fifty pills of twenty or twenty-five centigrammes each. Of these he gives at first, one a day, increasing gradually to five a day. The limit of toleration of the remedy is often indicated by frequent alvine discharges. Meat and eggs are useful for the phosphorous contained in them. Wine, coffee and cold douches to the head serve as valuable stimulants. The author also recommends mild mental gymnastics.—*Gazette Medico Italiana, Lombordia.*—*New York Medical Journal, January,* 1873.

*Treatment of Cancrum Oris.*—Dr. McGregory, in British Medical Journal says: "Of all the local remedies or applications that I have ever resorted to in the treatment of this affection, I have never found any remedy so useful or so effectual as hydrochloric acid. I have almost never known it to fail to check the progress of this dreadful disease at once, and bring on a most rapid and healthful action in the part; it causes but very little pain, as the gangrenous spot is almost without feeling at this time. It is easily applied with a feather or small camel-hair pencil."

*The Treatment of Prurigo and Prutis by Carbolic Acid.*—Dr. Rothmund (Œrlztl Intelligenzblatt, 39, 1872,) states that the internal administration of carbolic acid in pruritus excels every other method. He has tried the hypodermic administration of it with marked success, there being no local irritation produced, as one would expect beforehand.

*Phosphorous in Neuralgia.*—Dr. S. M. Bradley, "*Lancet,*" speaks highly of the use of $\frac{1}{20}$ grain doses of phosphorous in neuralgia, and cites several cases thus treated by him, and with marked success, cases being relieved by this, which had resisted every other remedy. It is worthy of a trial.

